# Associations of single nucleotide polymorphisms with mucinous colorectal cancer: genome-wide common variant and gene-based rare variant analyses

**DOI:** 10.1186/s40364-018-0133-z

**Published:** 2018-06-13

**Authors:** Michelle E. Penney, Patrick S. Parfrey, Sevtap Savas, Yildiz E. Yilmaz

**Affiliations:** 1 Discipline of Genetics, Faculty of Medicine, Memorial University of Newfoundland, St. John’s, Canada; 2 Discipline of Medicine, Faculty of Medicine, Memorial University of Newfoundland, St. John’s, Canada; 3 Discipline of Oncology, Faculty of Medicine, Memorial University of Newfoundland, St. John’s, Canada; 4 Department of Mathematics and Statistics, Faculty of Science, Memorial University of Newfoundland, St. John’s, Canada

**Keywords:** Colorectal cancer, Mucinous adenocarcinoma, Genome-wide association study, Common single nucleotide polymorphisms, Rare single nucleotide polymorphisms

## Abstract

**Background:**

Colorectal cancer has significant impact on individuals and healthcare systems. Many genes have been identified to influence its pathogenesis. However, the genetic basis of mucinous tumor histology, an aggressive subtype of colorectal cancer, is currently not well-known. This study aimed to identify common and rare genetic variations that are associated with the mucinous tumor phenotype.

**Methods:**

Genome-wide single nucleotide polymorphism (SNP) data was investigated in a colorectal cancer patient cohort (*n* = 505). Association analyses were performed for 729,373 common SNPs and 275,645 rare SNPs. Common SNP association analysis was performed using univariable and multivariable logistic regression under different genetic models. Rare-variant association analysis was performed using a multi-marker test.

**Results:**

No associations reached the traditional genome-wide significance. However, promising genetic associations were identified. The identified common SNPs significantly improved the discriminatory accuracy of the model for mucinous tumor phenotype. Specifically, the area under the receiver operating characteristic curve increased from 0.703 (95% CI: 0.634–0.773) to 0.916 (95% CI: 0.873–0.960) when considering the most significant SNPs. Additionally, the rare variant analysis identified a number of genetic regions that potentially contain causal rare variants associated with the mucinous tumor phenotype.

**Conclusions:**

This is the first study applying both common and rare variant analyses to identify genetic associations with mucinous tumor phenotype using a genome-wide genotype data. Our results suggested novel associations with mucinous tumors. Once confirmed, these results will not only help us understand the biological basis of mucinous histology, but may also help develop targeted treatment options for mucinous tumors.

**Electronic supplementary material:**

The online version of this article (10.1186/s40364-018-0133-z) contains supplementary material, which is available to authorized users.

## Background

Colorectal cancer is a global health problem and contributes substantially to worldwide cancer mortality [1]. In 2012, this disease was the 3rd most common cancer worldwide with higher rates occurring in developed countries [1]. In Canada, colorectal cancer is expected to cause 26,800 new cases and 9400 deaths in 2017. Newfoundland and Labrador, in particular, have the highest age-standardized rates of incidence and mortality in the country [2].

Mucins are a family of high-molecular-weight glycoproteins that are widely expressed by epithelial tissues [3]. According to the HGNC database [4], there are 22 members in this family that can be expressed in various tissues. They have been identified in two forms: cell surface (transmembrane), such as MUC1 and MUC4, and fully released (gel-forming) [3, 5, 6]. The gel-forming mucin-encoding genes are clustered at chromosome 11p15.5 [5, 7, 8]. These mucins, including MUC2, MUC5AC, MUC5B, and MUC6, constitute the major macromolecular components of mucus [5, 7, 9]. Among them, MUC2 is the most highly expressed one in the colorectum and is the predominant component of colorectal mucus [10–12]. MUC5B and MUC6 are highly expressed in the upper gastrointestinal (GI) tract, but low levels of both have been reported in the normal colon [12, 13]. MUC5AC is highly expressed in the upper GI tract and is not expressed in the normal colon, however, abnormal expression is observed in colorectal cancer [14–16].

Mucinous adenocarcinoma is a distinct form of colorectal cancer with the defining characteristic of a high mucin component (more than 50% of the tumor volume). This subtype accounts for 5–15% of colorectal cancer cases. Compared to non-mucinous colorectal cancer, mucinous adenocarcinoma patients are typically younger and are often at an advanced stage at diagnosis [17–23]. Mucinous tumors are more likely to occur in the proximal colon [20, 21, 24, 25] and tend to have an inferior response to systemic therapies [25, 26].

Specific molecular distinctions are also seen in mucinous compared to non-mucinous colorectal tumors, for example, increased rates of *BRAF* mutations and CpG island methylator phenotype (CIMP) [27]. In addition, overexpression of MUC2, strong ectopic expression of gastric MUC5AC, and decreased p53 expression in mucinous tumors are reported in the literature [28, 29]. Mucinous and non-mucinous tumors also appear to have differences in genome-wide gene expression patterns [23]. Some of the upregulated genes in mucinous tumors are involved in cellular differentiation and mucin metabolism, which are characteristics biologically relevant to the phenotype [23]. While the differences between mucinous and non-mucinous colorectal cancers are well recognized, the prognostic importance of a high mucin component has been controversial [19–21, 25, 26, 30–35].

Most studies investigating characteristics of mucinous colorectal tumors examined single or a limited number of candidate genes [10, 36, 37]. This study aimed to comprehensively identify common and rare genetic polymorphisms that may be influencing the production of mucin or formation of the mucinous tumor phenotype. To do so, we applied a genome-wide approach to identify genes and genetic regions that are associated with the risk of developing the mucinous tumor phenotype.

## Methods

### Patient cohort

The study cohort was a subgroup of the Newfoundland Colorectal Cancer Registry (NFCCR) and consisted of 505 Caucasian patients. Both the NFCCR and the study cohort were described in detail in other publications [38, 39]. In short, the NFCCR recruited 750 colorectal cancer patients in Newfoundland and Labrador collected between 1999 and 2003. All diagnoses were confirmed by pathological examination. Out of 750 patients, 505 patients constituted the study cohort as explained below.

### Genotype data

The genotype data used in this study was explained in Xu et al. (2015) [39]. In short, DNA samples of 539 patients were subject to whole-genome single nucleotide polymorphism (SNP) genotyping using the Illumina Omni1-Quad human SNP genotyping platform (Centrillion Bioscience, USA). These patients were included into the genetic analysis because of the availability of their outcome and clinical data as well as the germline DNAs extracted from peripheral blood samples. The quality control analysis and filtering for this data included removing SNPs whose frequencies deviated from Hardy-Weinberg equilibrium, SNPs that had >5% missing values, and patients with discordant sex information, accidental duplicates, divergent or non-Caucasian ancestry, and first, second, or third degree relatives [39]. In Xu et al. (2015) [39], 505 patients were examined to investigate associations between overall and disease-free survival times after colorectal cancer diagnosis and genetic polymorphisms with a minor allele frequency (MAF) of at least 5%. In our study, there were 505 patients with 729,373 common SNPs (MAF ≥0.05) and 275,645 rare SNPs (MAF <0.05) that were included. No SNP was excluded due to high or perfect linkage disequilibrium (LD) with other SNPs. During this study, management and handling of these genotype data was done using PLINK v. 1.07 [40].

### Statistical analysis

The response variable is a binary variable indicating existence of mucinous tumor histology or non-mucinous tumor histology.

### Common SNP analysis

#### Univariable logistic regression analysis

Univariable logistic regression analysis was performed on each common SNP (MAF ≥5%) to determine if individual SNPs were significantly associated with mucinous tumor phenotype (i.e. mucinous versus non-mucinous tumor histology). For each SNP, the additive, co-dominant, dominant, and recessive genetic models were applied. Consequently, we report the 10 SNPs without excluding those in high LD with the highest level of significance in each genetic model (Additional file 1: Tables S1-S4).

#### Selection of baseline variables and multivariable logistic regression analysis

In order to select significant baseline factors to adjust for in the multivariable analyses, we first examined the variables shown in Table 1 using univariable logistic regression models. These variables were selected for inclusion into the selection process based on previous studies investigating mucinous colorectal tumors [27, 33]. Factors that had a *p*-value less than 0.1 were then included in a forward stepwise variable selection method. In addition, although there appeared to be a non-significant association between tumor histology and grade in the univariable analysis, tumor grade was still included in the multivariable model as has been shown to be linked to tumor histology [30, 41]. As a result, the baseline characteristics in the final models were sex, age at diagnosis, stage, and tumor location based on the 0.1 level of significance, and tumor grade (Additional file 1: Table S5). The 10 SNPs with the highest level of significance under each genetic model in the univariable logistic regression analysis were analyzed using the multivariable logistic regression model adjusting for the selected baseline characteristics (Additional file 1: Tables S1-S4).

**Table 1:** Baseline features of the study cohort and the results of univariable logistic regression analysis

		Mucinous	Non-mucinous		
Characteristics		No. (%)	No. (%)	OR (95% CI)	*p*-value
Age^a^	≤60	20 (9)	203 (91)		
	60–65	17 (18)	78 (82)	2.21 (1.09–4.44)	0.025
	>65	20 (11)	167 (89)	1.22 (0.63–2.34)	0.558
Sex	Female	29 (15)	169 (85)		
	Male	28 (9)	279 (91)	0.58 (0.34–1.02)	0.057
Location	Colon	47 (14)	287 (86)		
	Rectum	10 (6)	161 (94)	0.38 (0.18–0.74)	0.007
Stage	I	3 (3)	90 (97)		
	II	27 (14)	169 (86)	4.79 (1.64–20.45)	0.012
	III	19 (11)	147 (89)	3.88 (1.28–16.83)	0.033
	IV	8 (16)	42 (84)	5.71 (1.57–27.09)	0.013
Grade	Well/moderately diff.	48 (10)	416 (90)		
	Poorly diff.	7 (19)	30 (81)	2.02 (0.78–4.62)	0.115
	Unknown	2	2		
MSI status	MSI-low/MSS	49 (11)	382 (89)		
	MSI-high	6 (11)	47 (89)	1.00 (0.37–2.29)	0.992
	Unknown	3	18		
Lymphatic invasion	Absent	31 (10)	267 (90)		
	Present	23 (14)	144 (86)	1.38 (0.77–2.44)	0.278
	Unknown	3	37		
*BRAF* V600E mutation	Absent	45 (11)	366 (89)		
	Present	9 (19)	38 (81)	1.93 (0.83–4.09)	0.104
	Unknown	3	44		

^a^The age at diagnosis was separated into 3 groups: ≤60, 60–65, and > 65 since the odds ratio does not remain constant for each year increase in age at diagnosis under the logistic regression model and this particular grouping gave the most efficient odds ratio estimates with no significant change in the results when considering slightly different groupings. *CI* confidence interval, *diff*. Differentiated, *MSI* microsatellite instability, *MSS* microsatellite stable, *No* number, *OR* odds ratio (compares the odds of having mucinous tumors with the corresponding factor level to the odds of having mucinous tumors with the reference factor level)

#### Plausibility of the genetic models

It is common in genetic association studies that only one genetic model is applied. In this study, we applied all four genetic models and assessed the plausibility of the genetic model under which the SNP was identified. To do this, we used the Akaike Information Criterion (AIC) calculations to compare the fit of four different genetic models per SNP under the multivariable logistic regression model. The genetic model with the smallest AIC estimate was considered to be the most plausible genetic model (i.e. the best fitting model). We first ranked the SNPs based on their *p*-value obtained in the multivariable model with the genetic model under which the SNP was identified (Additional file 1: Table S6). Then, we excluded those SNPs that were not identified in their plausible genetic model. Of note, we present in this manuscript only the 10 SNPs that have the highest association significance levels under the multivariable logistic regression models that were identified in their most plausible genetic model. We refer to these SNPs as “the top 10 SNPs”. The LD between SNPs was not taken into account when listing the top 10 SNPs.

#### Assessing the discriminatory accuracy of the estimated models

We aimed to check the ability of the multivariable models of the top 10 SNPs to discriminate between mucinous and non-mucinous phenotypes. A well-known method for assessing the discriminatory accuracy of a model is using a receiver operating characteristic (ROC) curve [42–44]. Calculating the area under the curve (AUC) of the ROC curve for the given models provides a single numeric representation for the performance of the model [43, 45, 46]. Comparing the AUC values and their corresponding confidence intervals provides a method for determining if one model is significantly superior to another in discriminatory accuracy [44, 47].

ROC curve analysis was performed by calculating the AUC using the pROC package in R [48]. The AUC estimates for (i) the model conditioning only on the baseline characteristics, (ii) the model conditioning on only the top SNPs, and (iii) the model conditioning on the baseline characteristics and the top SNPs. Comparing the AUC, specifically the 95% confidence intervals, between these three models can quantify the differences in the capacity of the models to distinguish mucinous and non-mucinous phenotypes.

### Rare variant analysis

#### SKAT-O analysis

SKAT-O [49] test statistic was used to test the associations between the rare variants and the mucinous tumor phenotype. For this analysis, we prioritized gene-based regions including 5 kb long sequences before and after each gene. To do so, we first obtained genome location information for genome-wide gene-based regions (for the reference genome GRCh37.p13) using the biomaRt tool [50] in the Ensembl database [51]. The SNP information within these regions were then retrieved from the patient genome-wide data and used as the region-based SNP-sets in SKAT-O. During this analysis, each SNP was assigned to one gene-based region only. As a result, when a gene is located in close proximity to another gene, the second gene-based region does not include the SNPs that are analyzed in the first gene-based region. This limits redundancy since no SNP is analyzed more than once. For this analysis, only the additive genetic model was considered as using multiple genetic models is not a practical option for SKAT-O. The associations of gene regions were examined in multivariable models, adjusting for the significant baseline characteristics sex, age at diagnosis, stage, tumor location, and tumor grade.

All statistical analysis was performed using R v. 3.1.3 [52]. Correction for multiple testing was not applied to the results as this is an exploratory study and we did not want to increase false negative rate due to conservative corrections. While this increases the chances of obtaining false positives, we believe replication of these results in other studies will assist in reducing the potential false positive findings.

### Bioinformatics analysis

Potential regulatory consequences of the identified SNPs were examined through RegulomeDB (http://www.regulomedb.org/) [53]. Ensembl [51] database was used to retrieve information related to the genes identified in the common and rare variant analysis.

## Results

The demographic and clinicopathological information for the sample population is shown in Table 1. We observed a non-significant association of histology with age at diagnosis (>65 versus ≤60), grade, microsatellite instability (MSI) status, lymphatic invasion (LI), and *BRAF* V600E mutation; a moderately significant association with stage, sex, and age at diagnosis between 60 and 65 versus ≤60; and a strongly significant association with tumor location (Table 1). In this cohort, there was a trend for female sex having increased risk of mucinous tumors. As expected, the proportion of mucinous tumors was higher in colon cancer patients compared to rectum cancer patients and in stage II-IV patients compared to stage I patients (Table 1).

### Common SNP analysis

None of the associations in this analysis reached the traditional genome-wide significance level (*P* < 5 × 10^−8^), but each genetic model identified promising associations.

After the univariable analysis, there were 33 SNPs that were nominally associated with the mucinous tumor phenotype (Additional file 1: Tables S1-S4)**.** Associations of two SNPs (rs11216624 & rs17712784) were identified in both the dominant and co-dominant genetic models; one SNP (rs7314811) was detected in the additive, recessive, and co-dominant genetic models; and three SNPs (rs4843335, rs10511330, & rs16822593) were detected in both the additive and dominant genetic models. The estimates obtained in the univariable analysis did not change significantly when the models were adjusted for the baseline characteristics (Additional file 1: Tables S1-S4).

As explained in the Methods section, the AIC estimates (Additional file 1: Table S6) were used to determine the most plausible genetic models for each of 33 SNPs. Ten SNPs with the smallest *p*-value in the multivariable analysis under the most plausible genetic models were further prioritized (i.e., the top 10 SNPs). The results of the univariable and the multivariable logistic regression analyses for these top 10 SNPs are summarized in Table 2. Seven of these SNPs were located within gene sequences. These genes were quite diverse and belong to a variety of biological processes and pathways (Table 3).

**Table 2:** Top ten promising SNPs identified in univariable analysis and the subsequent multivariable analysis under their plausible genetic models

					Univariable		Multivariable^e^	
Genomic location	SNP ID (Genotype^a^)	Gene^b^	Information in RegulomeDB	Plausible model^c^	OR (95% CI)	*p*-value	OR (95% CI)	*p*-value
Chr6:110750552	rs9481067 (GG)	*SLC22A16*	ND	Recessive	4.17 (2.33–7.43)	1.24E-06	4.75 (2.53–8.95)	1.24E-06
Chr3:114121019	rs10511330 (CT + CC)	*ZBTB20*	Minimal binding evidence	Dominant	3.77 (2.06–6.81)	1.24E-05	4.85 (2.54–9.23)	1.40E-06
Chr3:114117327	rs16822593 (AG + AA)	*ZBTB20*	ND	Dominant	3.70 (2.02–6.68)	1.59E-05	4.83 (2.53–9.20)	1.50E-06
Chr2:179860562	rs13019215 (TC + TT)	*CCDC141*	ND	Dominant	0.27 (0.14–0.48)	1.56E-05	0.23 (0.12–0.43)	8.20E-06
Chr2:179867985	rs12471607 (TC + TT)	*CCDC141*	ND	Dominant	0.27 (0.14–0.48)	1.65E-05	0.23 (0.12–0.43)	8.42E-06
Chr5:80483574	rs716897 (CT + CC)	*RASGRF2*	Minimal binding evidence	Dominant	0.27 (0.15–0.47)	5.33E-06	0.26 (0.14–0.47)	1.12E-05
Chr16:86077637	rs4843335 (AG + AA)	intergenic	Minimal binding evidence	Dominant	4.11 (2.11–7.79)	2.06E-05	4.67 (2.98–9.34)	1.48E-05
Chr6:118634698	rs11968293 (CA + CC)	*SLC35F1*	Minimal binding evidence	Dominant	0.28 (0.16–0.50)	1.27E-05	0.26 (0.14–0.48)	1.48E-05
Chr9:131923949	rs4837345 (TT)	intergenic	Minimal binding evidence	Recessive	4.72 (2.40–9.05)	4.00E-06	4.56 (2.24–9.11)	1.97E-05
Chr9:131930494	kgp10457679/ rs10819474^d^ (CC)	intergenic	Likely to affect binding and linked to expression of a gene target	Recessive	4.72 (2.40–9.05)	4.00E-06	4.56 (2.24–9.11)	1.97E-05

^a^Risk increasing/decreasing genotype. ^b^Based on Ensembl [51] or dbSNP databases [61]. ^c^Under the recessive genetic model, minor allele homozygous patients are compared to major allele homozygous and heterozygous patients combined. Under the dominant genetic model, minor allele homozygous and heterozygous patients are combined and compared to major allele homozygous patients. ^d^The rs number for the kgp10457679 polymorphism was obtained from the UCSC genome browser [62]. ^e^Multivariable models adjusted for sex, age at diagnosis, stage, tumor location, and tumor grade. Patients with missing/unknown data for any of these variables were excluded from the analysis. *Chr* chromosome, *CI* confidence interval, *ND* data not available at RegulomeDB, *OR* odds ratio (compares the odds of having mucinous tumors with the specified genotype(s)^a^ to the odds of having mucinous tumors with the reference (other) genotype(s))

**Table 3:** Genes identified in the common and rare analyses

Gene symbol^a^	Gene name^b^	Function
*SLC22A16*	solute carrier family 22 member 16	codes for a human ʟ-carnitine transporter protein hCT2. hCT2 has been shown to have undetectable expression in a colon cancer cell line. [63, 64]
*CCDC141*	coiled-coil domain containing 141	codes for a protein that plays a critical role in centrosome positioning and movement, particularly radial migration. Centrosome aberrations have been shown to be present in early-stage colorectal cancers and could contribute to chromosomal instability. [65, 66]
*SLC35F1*	solute carrier family 35 member F1	codes for a member of the solute carrier family 35, a family of nucleotide sugar transporters. [67]
*ZBTB20*	zinc finger and BTB domain containing 20	codes for a transcriptional repressor. Upregulation of ZBTB20 has been shown to promote cell proliferation in non-small cell lung cancer and is a potential druggable target for the disease. Similarly, overexpression of ZBTB20 has been associated with poor prognosis in patients with hepatocellular carcinoma. [68–70]
*RASGRF2*	Ras protein specific guanine nucleotide releasing factor 2	codes for a signalling molecule. RasGRF2 contains regulatory domains for both Ras and Rho GTPases, suggesting it can influence both pathways. The Rho pathway has been thought to be involved in cell migration, while the Ras pathway has been thought to be involved in cell proliferation and survival, which are all processes related to cancer. [71, 72]
*SEC24B*	SEC24 homolog B, COPII coat complex component	codes for a protein that is a part of the COPII vesicle coat, facilitating molecular transport from the endoplasmic reticulum to the Golgi apparatus. It has been suggested that alterations in vesicle trafficking proteins may be facilitators of epithelial carcinogenesis. [73, 74]
*CCDC109b*	coiled-coil domain containing 109B	also known as *MCUb*. This gene codes a protein that interacts with the mitochondrial calcium transporter protein, CCDC109a/MCU, reducing the activity of the transporter. Calcium homeostasis in mitochondria may regulate cell death pathways. [75, 76]
*LINC00596*	long intergenic non-protein coding RNA 596	no literature data available.
*SEC24B-AS1*	SEC24B antisense RNA 1	long non-coding RNA (lncRNA) that is involved in gene expression regulation. [77]
*RP11-564A8.8*	NA	no literature data available.
*FAM87A*	family with sequence similarity 87 member A	no literature data available.

^a^According to Ensembl database [51]. ^b^According to HUGO Gene Nomenclature Committee (HGNC) [4]. *NA* Not available

Before the ROC analysis, the LD among the top 10 SNPs were assessed using patient genotype data. These calculations indicated that rs13019215 and rs12471607 were in complete pairwise LD (r^2^ = 1). The SNPs rs4837345 and kgp10457679 were also in high LD with each other, as well as rs10511330 and rs16822593 (0.99 ≤ r^2^ ≤ 1.0). Therefore, we kept one SNP per SNP set in high LD, which left the following SNPs for the ROC analysis: rs9481067, rs10511330, rs13019215, rs716897, rs4843335, rs11968293, and kgp10457679.

**Fig. 1:**
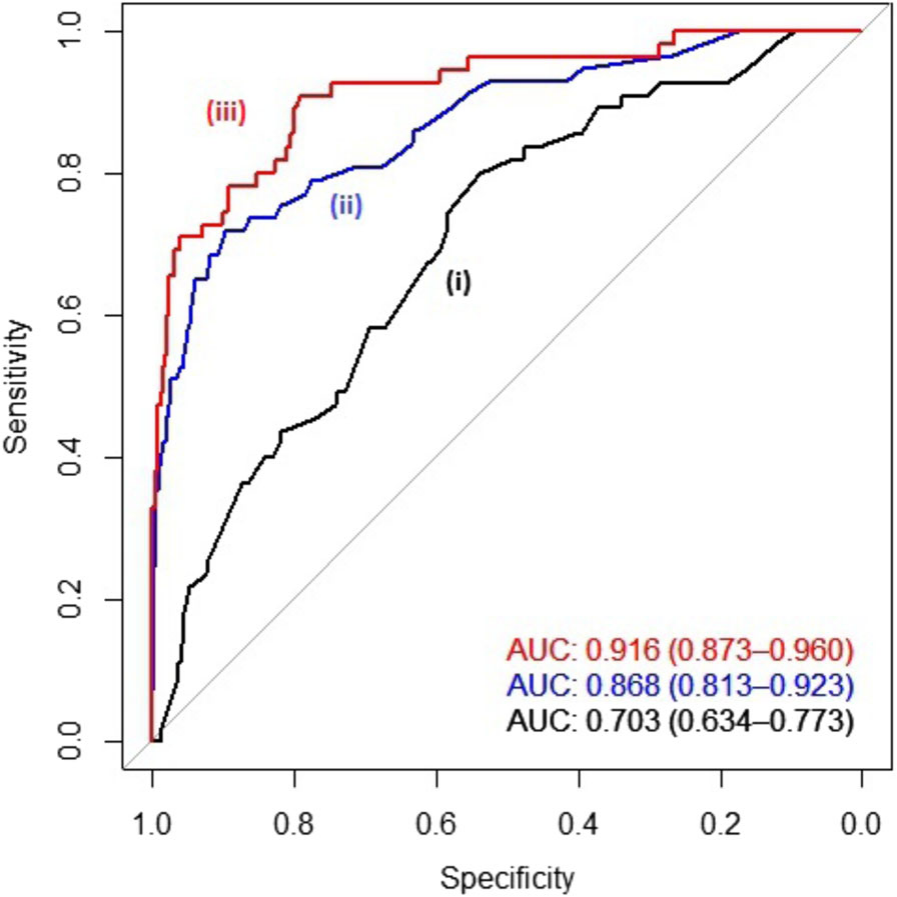
ROC curves and corresponding AUC values for multivariable models. Due to high LD among some of the top 10 SNPs, ROC analysis was performed on only the following SNPs: rs9481067, rs10511330, rs13019215, rs716897, rs4843335, rs11968293, and kgp10457679. AUC: area under the ROC curve, CI: confidence interval, LD: linkage disequilibrium, ROC: receiver operator characteristic

Figure 1 shows the ROC curves comparing the accuracy of the models to discriminate mucinous and non-mucinous tumor phenotypes. The model (iii) including both the baseline characteristics and the SNPs (AUC = 0.916, CI: 0.873–0.960) had the most discriminatory accuracy followed by model (ii) including only the SNPs (AUC = 0.868, CI: 0.813–0.923) and model (i) including only the baseline characteristics (AUC = 0.703, 95% CI: 0.634–0.773). Since the confidence intervals of models (i) and (iii) do not overlap, we can confidently claim that there is a statistically significant improvement in the discriminating accuracy of the model containing the SNPs [44, 47]. This also suggests that these SNPs explain some of the variation between the mucinous and non-mucinous tumor phenotypes.

### Rare SNP analysis

In the gene region-based rare variant analysis, we investigated 29,966 regions in the patient cohort using the multivariable SKAT-O method. Table 3 and Table 4 summarize the most significant regions (*P* < 10^−4^) that potentially contain causal rare variants associated with the mucinous tumor phenotype. The number of variants aggregated in these gene-based regions varied from 5 to 10. While three of these regions (including the *SEC24B*, *SEC24B-AS1*, and *CCDC109B* regions) were located close to each other on chromosome 4, other regions come from different parts of the genome (Table 4).

**Table 4:** Most significant gene regions identified from SKAT-O multivariable analysis

Genomic location^a^	Gene^b^	Description^c^	Other genes in the gene-based region^d^	# of SNPs	SNPs	*P*-value
Chr4:110349928–110467052	*SEC24B*	protein coding	*SEC24B-AS1* (partial sequence)	5	rs10516557, kgp21293502, rs10003981, rs17040515, rs17040519	1.81E-05
Chr4:110476361–110614874	*CCDC109B*	protein coding	*CDC42P4* (pseudogene: partial sequence), *HIGD1AP14* (pseudogene; full length), *CASP6* (partial sequence)	6	rs17619262, rs7654187, rs6831048, rs17619310, rs9997940, rs1053680	3.29E-05
Chr14:24386456–24408777	*LINC00596*	long intergenic non-protein coding RNA	*DHRS4-AS1* (partial sequence)	6	rs8010486, rs1159372, rs10135026, rs8005541, rs8019962, kgp19564619	3.34E-05
Chr4:110263631–110359973	*SEC24B-AS1*	noncoding RNA; antisense RNA	*RBMXP4* (pseudogene; full length), *SEC24B* (partial sequence)	7	rs10031399, rs17040364, rs17040369, rs11098033, rs17040401, rs12648138, rs11098035	4.21E-05
Chr1:207074273–207084738	*RP11-564A8.8*	pseudogene	*IL24* (partial sequence), *FAIM3* (partial sequence), *FCMR* (partial sequence)	10	rs3093428, kgp15249933, kgp15191074, rs3093447, kgp22852559, rs3093434, rs3093437, rs3093438, rs3093440, rs41304091	5.47E-05
Chr8:320931–338174	*FAM87A*	non-coding RNA	–	7	rs4527844, kgp20525414, kgp20198205, rs11785854, rs7461388, rs17064450, rs17064458	6.58E-05

^a^These genomic locations describe the region containing the gene as well as 5 kb long sequences before and after the gene. ^b^Based on the information in the UCSC database [62]. ^c^NCBI’s Gene Entrez database [77]. ^d^In some cases, the gene regions examined also contained sequences of other genes. *Chr* chromosome

## Discussion

Mucinous tumors are considered an aggressive type of colorectal tumors that are poorly understood [22, 24, 54]. While their role in prognosis is not well established, several studies suggested these tumors are associated with poorer prognosis when compared to non-mucinous tumors [25, 26, 32, 33, 35]. Identification of genes and genetic variations that can have a role in mucinous tumor development, therefore, has both scientific (e.g. dissecting the biology behind the mucinous tumor histology) as well as clinical value (e.g. biological information gained may assist with development of targeted treatment for this cancer subtype). Accordingly, for the first time with this study, we examined associations of both common and rare variants with the risk of developing the mucinous tumor phenotype using a genome-wide dataset.

While our results did not reach the conservative genome-wide significance level, promising associations were detected in both the common and rare variant analyses. In common SNP analysis, we identified seven unlinked polymorphisms that significantly increased our capacity to discriminate between mucinous and non-mucinous tumor phenotypes (Fig. 1, Table 2). Their effects on tumor histology were independent from the effects of the baseline variables (Fig. 1, Table 2). It is possible these polymorphisms (or others in high LD with them (Additional file 1: Table S7), including three additional SNPs shown in Table 2) are biologically linked to tumor histology or mucin production. Since there was no reported functional consequence of these SNPs in the literature, we searched the RegulomeDB database [53] for their potential biological characteristics. As of March 2018, the only SNP with a predicted/reported regulatory function in this database was kgp10457679 (rs10819474) (RegulomeDB score = 1f). This intergenic SNP is categorized as an expression quantitative trait locus (eQTL)/Transcription Factor (TF) binding/DNAse peak site, with a likely role of influencing the expression of target genes (Additional file 1: Table S8). Specifically, *PPP2R4* is noted as the eQTL for this SNP. PPP2R4 is a tumor suppressor protein [55] which has been shown to have low activity in a large portion of a small cohort of colorectal tumors [56] and is associated with shorter survival times in metastatic colorectal cancer patients [57]. A potential link of PPP2R4 to mucinous tumor phenotype risk should be examined in further studies. Interestingly, one GWAS identified a SNP within the sequences of *ZBTB20*, other than the one reported in this study, that is significantly associated with the risk of non-cardia gastric cancer in the Han Chinese population [58]. Overall, all the novel loci identified by the common variant analysis are interesting candidates in examination of mucinous tumor development.

Typical association studies, such as the common variant analysis, focus on a variant-by-variant approach, which is underpowered for rare variants. It has been suggested that gene/region-based approaches can be useful in increasing the power under these circumstances where the direct effects of multiple variants on a phenotype can be examined [59]. Hence, in this study, we performed the first rare variant analysis to explore gene regions that may have a role in mucinous tumor formation using SKAT-O [49]. SKAT-O is a multi-marker association test which has reasonable type I error rate and is a powerful test under many scenarios [49]. In our study, this method identified a number of gene-based regions that may harbor rare variants associated with mucinous phenotype (Tables 3 and 4). Interestingly, three of the gene-based regions in Table 4 (*SEC24B*, *SEC24B-AS1*, and *CCDC109B*-based regions) were located in a 341,243 bp long genomic region on chromosome 4q. Since we assigned each SNP to only one gene region, these results suggest that these three gene regions are associated with the mucinous phenotype independent of each other. A search on the RegulomeDB database [53] indicated that one of the SNPs in *LINC00596* (rs8005541) could have a strong regulatory function (RegulomeDB score = 1f). This variant is located in an eQTL and seems to affect the expression of two nearby genes; *DHRS4* and *DHRS4L2*. These two genes are a part of a gene cluster on chromosome 14 that code for dehydrogenases/reductases [60] and have not been previously linked to mucinous tumors. Similarly, none of the genes in Table 4 had a previously identified connection to the risk of developing mucinous tumors. In conclusion, these regions, genes, or SNPs, alone or in combination, may be influential on the mucinous tumor phenotype and should be explored further.

Several strengths and limitations of this study should be mentioned. Studying the mucinous tumor phenotype is inherently challenging since it is not frequently detected. Despite this and the large number of SNPs/gene-based regions investigated, this study identified promising genetic variants and genomic regions that may have a biological connection to the mucinous tumor phenotype. We are aware that our results need to be replicated in independent cohorts and remain to be verified. Of note, SNPs and genetic regions we report are different than the MUC genes, which are the typical candidate genes for mucin production and mucinous phenotype. In the common variant analysis, the recessive and co-dominant models yielded some high odds ratio estimates but also wide confidence intervals (as expected, as these are the models with relatively low power). Consequently, the interpretation of these results should be made with caution. SKAT-O is a robust test and an attractive choice for rare variant analysis, however, it cannot determine which SNPs or how many SNPs within a SNP-set are truly associated with the phenotype. Also, in the rare variant analysis, due to the assignment of one SNP to one gene region, there could be some genes whose associations may have been missed. In addition, in contrast to previous studies, we used a comprehensive genome-wide SNP genotype data, however, analysis of a more comprehensive data (such as those obtained by whole genome sequencing) would be desirable. This is particularly true for rare variants as most genotyping technologies target primarily common SNPs.

## Conclusions

In this study, we performed the first genome-wide association study on common and rare SNPs in colorectal cancer patients to identify novel genetic associations with the mucinous tumor phenotype. We identified novel, promising, and independent associations of specific SNP genotypes with the risk of developing mucinous tumors. In the common and rare variant analysis, we reported SNPs within the sequences of genes encoding transporter proteins, such as SLC22A16 and SLC35F1, which may have a role in transporting molecules related to excessive mucin production. In addition, the rare variant analysis reported associations with several regulating RNA molecules, which may influence the expression of genes related to mucin production. Finally, the common SNP analysis reports genes whose protein products are involved in DNA replication (CCDC141) and transcription (ZBTB20) that could have downstream effects on the mucin genes. Furthermore, the common SNPs reported in this study significantly improved the discriminatory accuracy of the multivariable model to distinguish between mucinous and non-mucinous tumors. In addition, we detected novel promising associations between gene-based sets of rare SNPs and mucinous tumors. The results of this study, once replicated in other cohorts, can contribute further information to the molecular characteristics of this under-studied but clinically important colorectal cancer subtype.

## Additional file

Additional file 1: **Table S1.** Top ten most significant common SNPs identified based on the univariable analyses and the subsequent multivariable analyses under the additive genetic models. **Table S2.** Top ten most significant common SNPs identified based on the univariable analyses and the subsequent multivariable analysis under the dominant genetic models. **Table S3**. Top ten most significant common SNPs identified based on the univariable analyses and the subsequent multivariable analyses under the recessive genetic models. **Table S4.** Top ten most significant common SNPs identified under the univariable analyses and the subsequent multivariable analyses under the co-dominant genetic models. **Table S5.** Baseline characteristics selected through a stepwise variable selection method under the multivariable model. **Table S6.** AIC estimates under the multivariable models of common SNPs identified in the univariable analysis. **Table S7.** Haploreg results for the top 10 SNPs in the common variant analysis. **Table S8.** Proteins which have reported evidence of binding to the genomic region in which kgp10457679 resides (extracted from RegulomeDB). (PDF 360 kb)

## Abbreviations

AIC: Akaike information criterion
AUC: Area under the curve
CI: Confidence interval
DNA: Deoxyribonucleic acid
eQTL: Expression quantitative trait loci
GI: Gastrointestinal
LD: Linkage disequilibrium
LI: Lymphatic invasion
MAF: Minor allele frequency
MSI: Microsatellite instability
NFCCR: Newfoundland Colorectal Cancer Registry
ROC: Receiver operating characteristic
SNP: Single nucleotide polymorphism
TF: Transcription factor
USA: United States of America

## References

[1] Ferlay J, Soerjomataram I, Dikshit R, Eser S, Mathers C, Rebelo M (2015). Cancer incidence and mortality worldwide: sources, methods and major patterns in GLOBOCAN 2012. Int J Cancer.

[2] Canadian Cancer Society’s Advisory Committee on Cancer Statistics (2017). Canadian Cancer Statistics 2017.

[3] Moniaux N, Escande F, Porchet N, Aubert JP, Batra SK (2001). Structural organization and classification of the human mucin genes. Front Biosci.

[4] Gray KA, Yates B, Seal RL, Wright MW, Bruford EA (2014). Genenames.org: the HGNC resources in 2015. Nucleic Acids Res.

[5] Desseyn J, Aubert J, Porchet N, Laine A (2000). Evolution of the large secreted gel-forming mucins. Mol Biol Evol.

[6] Dhanisha SS, Guruvayoorappan C, Drishya S, Abeesh P (2018). Mucins: structural diversity, biosynthesis, its role in pathogenesis and as possible therapeutic targets. Crit Rev Oncol Hematol.

[7] Desseyn J, Buisine M, Porchet N, Aubert J, Degand P, Laine A (1998). Evolutionary history of the 11p15 human mucin gene family. J Mol Evol.

[8] Gosalia N, Leir S, Harris A (2012). Coordinate regulation of the gel-forming mucin genes at chromosome 11p15.5. J Biol Chem.

[9] Corfield AP (2015). Mucins: a biologically relevant glycan barrier in mucosal protection. Biochim Biophys Acta Gen Sub.

[10] Okudaira K, Kakar S, Cun L, Choi E, Wu Decamillis R, Miura S (2010). MUC2 gene promoter methylation in mucinous and non-mucinous colorectal cancer tissues. Int J Oncol.

[11] Johansson MEV, Larsson JMH, Hansson GC (2010). The two mucus layers of colon are organized by the MUC2 mucin, whereas the outer layer is a legislator of host-microbial interactions. Proc Natl Acad Sci U S A.

[12] Ho SB, Niehans GA, Lyftogt C, Yan PS, Cherwitz DL, Gum ET (1993). Heterogeneity of mucin gene expression in normal and neoplastic tissues. Cancer Res.

[13] Toribara NW, Roberton AM, Ho SB, Kuo WL, Gum E, Hicks JW (1993). Human gastric mucin. Identification of a unique species by expression cloning. J Biol Chem.

[14] Biemer-Hüttmann A, Walsh MD, McGuckin MA, Ajioka Y, Watanabe H, Leggett BA (1999). Immunohistochemical staining patterns of MUC1, MUC2, MUC4, and MUC5AC mucins in hyperplastic polyps, serrated adenomas, and traditional adenomas of the colorectum. J Histochem Cytochem.

[15] Bartman AE, Serson SJ, Ewing SL, Niehans GA, Wiehr CL, Evans MK (1999). Aberrant expression of MUC5AC and MUC6 gastric mucin genes in colorectal polyps. Int J Cancer.

[16] Amini A, Masoumi-Moghaddam S, Ehteda A, Liauw W, Morris DL (2015). Depletion of mucin in mucin-producing human gastrointestinal carcinoma: results from in vitro and in vivo studies with bromelain and N-acetylcysteine. Oncotarget.

[17] Wu C, Tung S, Chen P, Kuo Y (1996). Clinicopathological study of colorectal mucinous carcinoma in Taiwan: a multivariate analysis. J Gastroenterol Hepatol.

[18] Odone V, Chang L, Caces J, George SL, Pratt CB (1982). The natural history of colorectal carcinoma in adolescents. Cancer.

[19] Chew M, Yeo SE, Ng Z, Lim K, Koh P, Ng K (2010). Critical analysis of mucin and signet ring cell as prognostic factors in an Asian population of 2,764 sporadic colorectal cancers. Int J Color Dis.

[20] Papadopoulos VN, Michalopoulos A, Netta S, Basdanis G, Paramythiotis D, Zatagias A (2004). Prognostic significance of mucinous component in colorectal carcinoma. Tech Coloproctol.

[21] Kang H, O'Connell BJ, Maggard AM, Sack J, Ko YC (2005). A 10-year outcomes evaluation of mucinous and signet-ring cell carcinoma of the colon and rectum. Dis Colon Rectum.

[22] Consorti F, Lorenzotti A, Midiri G, Di Paola M (2000). Prognostic significance of mucinous carcinoma of colon and rectum: a prospective case-control study. J Surg Oncol.

[23] Melis M, Hernandez J, Siegel EM, McLoughlin JM, Ly QP, Nair RM (2010). Gene expression profiling of colorectal mucinous adenocarcinomas. Dis Colon Rectum.

[24] Nozoe T, Anai H, Nasu S, Sugimachi K (2000). Clinicopathological characteristics of mucinous carcinoma of the colon and rectum. J Surg Oncol.

[25] Catalano V, Loupakis F, Graziano F, Torresi U, Bisonni R, Mari D (2009). Mucinous histology predicts for poor response rate and overall survival of patients with colorectal cancer and treated with first-line oxaliplatin- and/or irinotecan-based chemotherapy. Br J Cancer.

[26] Negri FV, Wotherspoon A, Cunningham D, Norman AR, Chong G, Ross PJ (2005). Mucinous histology predicts for reduced fluorouracil responsiveness and survival in advanced colorectal cancer. Ann Oncol.

[27] Tanaka H, Deng G, Matsuzaki K, Kakar S, Kim GE, Miura S (2006). BRAF mutation, CpG island methylator phenotype and microsatellite instability occur more frequently and concordantly in mucinous than non-mucinous colorectal cancer. Int J Cancer.

[28] Hanski C, Tiecke F, Hummel M, Hanski M, Ogorek D, Rolfs A (1996). Low frequency of p53 gene mutation and protein expression in mucinous colorectal carcinomas. Cancer Lett.

[29] Park SY, Lee HS, Choe G, Chung JH, Kim WH (2006). Clinicopathological characteristics, microsatellite instability, and expression of mucin core proteins and p53 in colorectal mucinous adenocarcinomas in relation to location. Virchows Arch.

[30] Farhat MH, Barada KA, Tawil AN, Itani DM, Hatoum HA, Shamseddine AI (2008). Effect of mucin production on survival in colorectal cancer: a case-control study. World J Gastroenterol.

[31] Nitsche U, Zimmermann A, Späth C, Müller T, Maak M, Schuster T (2013). Mucinous and signet-ring cell colorectal cancers differ from classical adenocarcinomas in tumor biology and prognosis. Ann Surg.

[32] Numata M, Shiozawa M, Watanabe T, Tamagawa H, Yamamoto N, Morinaga S (2012). The clinicopathological features of colorectal mucinous adenocarcinoma and a therapeutic strategy for the disease. World J Surg Oncol.

[33] Verhulst J, Ferdinande L, Demetter P, Ceelen W (2012). Mucinous subtype as prognostic factor in colorectal cancer: a systematic review and meta-analysis. J Clin Pathol.

[34] Nitsche U, Friess H, Agha A, Angele M, Eckel R, Heitland W (2016). Prognosis of mucinous and signet-ring cell colorectal cancer in a population-based cohort. J Cancer Res Clin Oncol.

[35] Park JS, Huh JW, Park YA, Cho YB, Yun SH, Kim HC (2015). Prognostic comparison between mucinous and nonmucinous adenocarcinoma in colorectal cancer. Medicine.

[36] Hanski C (1995). Is mucinous carcinoma of the colorectum a distinct genetic entity?. Br J Cancer.

[37] Kim DH, Kim JW, Cho JH, Baek SH, Kakar S, Kim GE (2005). Expression of mucin core proteins, trefoil factors, APC and p21 in subsets of colorectal polyps and cancers suggests a distinct pathway of pathogenesis of mucinous carcinoma of the colorectum. Int J Oncol.

[38] Woods MO, Hyde AJ, Curtis FK, Stuckless S, Green JS, Pollett AF (2005). High frequency of hereditary colorectal cancer in Newfoundland likely involves novel susceptibility genes. Clin Cancer Res.

[39] Xu W, Xu J, Shestopaloff K, Dicks E, Green J, Parfrey P (2015). A genome wide association study on Newfoundland colorectal cancer patients' survival outcomes. Biomarker Res.

[40] Purcell S, Neale B, Todd-Brown K, Thomas L, Ferreira M, Bender D (2007). PLINK: a tool set for whole-genome association and population-based linkage analyses. Am J Hum Genet.

[41] Leopoldo S, Lorena B, Cinzia A, Gabriella D, Angela Luciana B, Renato C (2008). Two subtypes of mucinous adenocarcinoma of the colorectum: clinicopathological and genetic features. Ann Surg Oncol.

[42] Lasko TA, Bhagwat JG, Zou KH, Ohno-Machado L (2005). The use of receiver operating characteristic curves in biomedical informatics. J Biomed Inform.

[43] Zhou X, Obuchowski NA, McClish DK (2011). Chapter 2. Measures of diagnostic accuracy. Statistical methods in diagnostic medicine. 2nd ed. Hoboken: Wiley.

[44] Søreide K (2008). Receiver-operating characteristic curve analysis in diagnostic, prognostic and predictive biomarker research. J Clin Pathol.

[45] Hanley JA, McNeil BJ (1982). The meaning and use of the area under a receiver operating characteristic (ROC) curve. Radiology.

[46] Kumar R, Indrayan A (2011). Receiver operating characteristic (ROC) curve for medical researchers. Indian Pediatr.

[47] Zweig MH, Campbell G (1993). Receiver-operating characteristic (ROC) plots: a fundamental evaluation tool in clinical medicine. Clin Chem.

[48] Robin X, Turck N, Hainard A, Tiberti N, Lisacek F, Sanchez J (2011). pROC: an open-source package for R and S+ to analyze and compare ROC curves. BMC Bioinformatics.

[49] Lee S, Wu MC, Lin X (2012). Optimal tests for rare variant effects in sequencing association studies. Biostatist.

[50] Kinsella RJ, Kähäri A, Haider S, Zamora J, Proctor G, Spudich G, et al. Ensembl BioMarts: a hub for data retrieval across taxonomic space. Database. 2011:bar030. 10.1093/database/bar030 PMC3170168 21785142

[51] Flicek P, Amode MR, Barrell D, Beal K, Billis K, Brent S (2014). Ensembl 2014. Nucleic Acids Res.

[52] Core Team R (2013). R: a language and environment for statistical computing. R Foundation for Statistical Computing.

[53] Boyle AP, Hong EL, Hariharan M, Cheng Y, Schaub MA, Kasowski M (2012). Annotation of functional variation in personal genomes using RegulomeDB. Genome Res.

[54] Yamamoto S, Mochizuki H, Hase K, Yamamoto T, Ohkusa Y, Yokoyama S (1993). Assessment of clinicopathologic features of colorectal mucinous adenocarcinoma. Am J Surg.

[55] Janssens V, Goris J, Van Hoof C (2005). PP2A: the expected tumor suppressor. Curr Opin Genet Dev.

[56] Cristóbal I, Rincón R, Manso R, Madoz-Gúrpide J, Caramés C, del Puerto-Nevado L (2014). Hyperphosphorylation of PP2A in colorectal cancer and the potential therapeutic value showed by its forskolin-induced dephosphorylation and activation. Biochim Biophys Acta Mol Basis Dis.

[57] Cristóbal I, Manso R, Rincón R, Caramés C, Zazo S, del Pulgar TG (2014). Phosphorylated protein phosphatase 2A determines poor outcome in patients with metastatic colorectal cancer. Br J Cancer.

[58] Shi Y, Hu Z, Wu C, Dai J, Li H, Dong J (2011). A genome-wide association study identifies new susceptibility loci for non-cardia gastric cancer at 3q13.31 and 5p13.1. Nat Genet.

[59] Li B, Leal SM (2008). Methods for detecting associations with rare variants for common diseases: application to analysis of sequence data. Am J Hum Genet.

[60] Gabrielli F, Tofanelli S (2012). Molecular and functional evolution of human DHRS2 and DHRS4 duplicated genes. Gene.

[61] Sherry ST, Ward MH, Kholodov M, Baker J, Phan L, Smigielski EM (2001). dbSNP: the NCBI database of genetic variation. Nucleic Acids Res.

[62] Kent WJ, Sugnet C,W., Furey TS, Roskin KM, Pringle TH, Zahler AM, et al. The human genome browser at UCSC. Genome Res 2002;12(6):996–1006. 10.1101/gr.229102 PMC186604 12045153

[63] Enomoto A, Wempe MF, Tsuchida H, Shin HJ, Cha SH, Anzai N (2002). Molecular identification of a novel carnitine transporter specific to human testis: insights into the mechanism of carnitine recognition. J Biol Chem.

[64] Aouida M, Poulin R, Ramotar D (2009). The human carnitine transporter SLC22A16 mediates high affinity uptake of the anticancer polyamine analogue bleomycin-A5. J Biol Chem.

[65] Fukuda T, Sugita S, Inatome R, Yanagi S (2010). CAMDI, a novel disrupted in schizophrenia 1 (DISC1)-binding protein, is required for radial migration. J Biol Chem.

[66] Kayser G, Gerlach U, Walch A, Nitschke R, Haxelmans S, Kayser K (2005). Numerical and structural centrosome aberrations are an early and stable event in the adenoma-carcinoma sequence of colorectal carcinomas. Virchows Arch.

[67] Ishida N, Kawakita M (2004). Molecular physiology and pathology of the nucleotide sugar transporter family (SLC35). Pflugers Arch.

[68] Xie Z, Zhang H, Tsai W, Zhang Y, Du Y, Zhong J (2008). Zinc finger protein ZBTB20 is a key repressor of alpha-fetoprotein gene transcription in liver. Proc Natl Acad Sci U S A.

[69] Zhao J, Ren K, Tang J (2014). Zinc finger protein ZBTB20 promotes cell proliferation in non-small cell lung cancer through repression of FoxO1. FEBS Lett.

[70] Wang Q, Tan Y, Ren Y, Dong L, Xie Z, Tang L (2011). Zinc finger protein ZBTB20 expression is increased in hepatocellular carcinoma and associated with poor prognosis. BMC Cancer.

[71] Fan W, Koch CA, de Hoog CL, Fam NP, Moran MF (1998). The exchange factor Ras-GRF2 activates Ras-dependent and Rac-dependent mitogen-activated protein kinase pathways. Curr Biol.

[72] Crespo P, Calvo F, Sanz-Moreno V (2011). Ras and rho GTPases on the move: the RasGRF connection. BioArchitecture.

[73] Wendeler MW, Paccaud J, Hauri H (2006). Role of Sec24 isoforms in selective export of membrane proteins from the endoplasmic reticulum. EMBO Rep.

[74] Goldenring JR (2013). A central role for vesicle trafficking in epithelial neoplasia: intracellular highways to carcinogenesis. Nat Rev Cancer.

[75] Raffaello A, De Stefani D, Sabbadin D, Teardo E, Merli G, Picard A (2013). The mitochondrial calcium uniporter is a multimer that can include a dominant-negative pore-forming subunit. EMBO J.

[76] Duchen MR (2000). Mitochondria and calcium: from cell signalling to cell death. J Physiol.

[77] Brown GR, Hem V, Katz KS, Ovetsky M, Wallin C, Ermolaeva O (2014). Gene: a gene-centered information resource at NCBI. Nucleic Acids Res.

[78] Green RC, Green JS, Buehler SK, Robb JD, Daftary D, Gallinger S (2007). Very high incidence of familial colorectal cancer in Newfoundland: a comparison with Ontario and 13 other population-based studies. Familial Cancer.

