# Supplementary material for: Associations of single nucleotide polymorphisms with mucinous colorectal cancer: genome-wide common variant and gene-based rare variant analyses
Source: Biomark Res. 2018 Jun 13;6:17. doi: 10.1186/s40364-018-0133-z (PMC5998544; doi:10.1186/s40364-018-0133-z)
Supplement: Supplementary file 1 — Table S1. Top ten most significant common SNPs identified based on the univariable analyses and the subsequent multivariable analyses under the additive genetic models. Table S2. Top ten most significant common SNPs identified based on the univariable analyses and the subsequent multivariable analysis under the dominant genetic models. Table S3. Top ten most significant common SNPs identified based on the univariable analyses and the subsequent multivariable analyses under the recessive genetic models. Table S4. Top ten most significant common SNPs identified under the univariable analyses and the subsequent multivariable analyses under the co-dominant genetic models. Table S5. Baseline characteristics selected through a stepwise variable selection method under the multivariable model. Table S6. AIC estimates under the multivariable models of common SNPs identified in the univariable analysis. Table S7. Haploreg results for the top 10 SNPs in the common variant analysis. Table S8. Proteins which have reported evidence of binding to the genomic region in which kgp10457679 resides (extracted from RegulomeDB). (PDF 360 kb) [file 40364_2018_133_MOESM1_ESM.pdf]

S1 Table. Top ten most significant common SNPs identified based on the univariable analyses and the subsequent multivariable analyses under the additive genetic models.

	Univariable		Multivariable*	
**SNP ID**	**OR (95% CI)**	**P-value**	**OR (95% CI)**	**P-value**
rs11159673 (AG + 2*AA vs. GG)	3.096 (1.879-5.073)	7.11E-06	3.016 (1.766-5.138)	4.53E-05
rs7314811 (CT + 2*CC vs. TT)	2.387 (1.611-3.567)	1.67E-05	2.129 (1.409-3.242)	3.59E-04
rs4843335 (AG + 2*AA vs. GG)	3.818 (2.040-7.037)	1.97E-05	4.160 (2.130-8.107)	2.64E-05
rs10511330 (CT + 2*CC vs. TT)	3.013 (1.788-5.031)	2.59E-05	3.908 (2.211-6.945)	2.65E-06
rs12915222 (CT + 2*TT vs. CC)	2.476 (1.623-3.801)	2.76E-05	2.664 (1.685-4.260)	3.19E-05
rs12956191 (GA + 2*GG vs. AA)	2.364 (1.580-3.549)	2.86E-05	2.397 (1.555-3.726)	8.18E-05
rs11648965 (GA + 2*GG vs. AA)	3.033 (1.786-5.104)	3.04E-05	3.250 (1.867-5.671)	2.82E-05
rs16822593 (AG + 2*AA vs. GG)	2.977 (1.767-4.968)	3.11E-05	3.899 (2.205-6.930)	2.78E-06
rs205536 (CT + 2*CC vs. TT)	2.494 (1.636-3.885)	3.28E-05	2.681 (1.717-4.294)	2.34E-05
rs2384298 (CT + 2*CC vs. TT)	2.332 (1.563-3.489)	3.42E-05	2.406 (1.576-3.702)	5.14E-05

CI: confidence interval, OR: odds ratio.
OR is the ratio of the odds of having mucinous tumors for one minor allele increase.
*Multivariable logistic regression model adjusting for the selected baseline characteristics listed in S5 Table.

S2 Table. Top ten most significant common SNPs identified based on the univariable analyses and the subsequent multivariable analysis under the dominant genetic models.

	Univariable		Multivariable*	
**SNP ID (a vs. b)**	**OR (95% CI)**	**P-value**	**OR (95% CI)**	**P-value**
rs716897 (CC + CT vs. TT)	0.27 (0.15-0.47)	5.33E-06	0.26 (0.14-0.47)	1.12E-05
rs10511330 (CC + CT vs. TT)	3.77 (2.06-6.81)	1.24E-05	4.85 (2.54-9.23)	1.40E-06
rs11968293 (CC + CA vs. AA)	0.28 (0.16-0.50)	1.27E-05	0.26 (0.14-0.48)	1.48E-05
rs17712784 (AA + AG vs. GG)	3.47 (1.97-6.12)	1.54E-05	3.30 (1.80-6.06)	1.12E-04
rs13019215 (TT + TC vs. CC)	0.27 (0.14-0.48)	1.56E-05	0.23 (0.12-0.43)	8.20E-06
rs16822593 (AA + AG vs. GG)	3.70 (2.02-6.68)	1.59E-05	4.83 (2.53-9.20)	1.50E-06
rs12471607 (TT + TC vs. CC)	0.27 (0.14-0.48)	1.65E-05	0.23 (0.12-0.43)	8.42E-06
rs4843335 (AA + AG vs. GG)	4.11 (2.11-7.79)	2.06E-05	4.67 (2.30-9.34)	1.48E-05
rs11216624 (AA + AG vs. GG)	3.56 (1.93-6.46)	3.34E-05	3.07 (1.60-5.79)	5.67E-04
rs9809129 (AA + AG vs. GG)	0.23 (0.11-0.45)	4.78E-05	0.23 (0.10-0.45)	6.52E-05

CI: confidence interval, OR: odds ratio.
OR compares the odds of having mucinous tumors in subgroup a to the odds of having mucinous tumors in subgroup b.
*Multivariable logistic regression model adjusting for the selected baseline characteristics listed in S5 Table.

S3 Table. Top ten most significant common SNPs identified based on the univariable analyses and the subsequent multivariable analyses under the recessive genetic models.

	Univariable		Multivariable*	
**SNP ID (a vs. b)**	**OR (95% CI)**	**P-value**	**OR (95% CI)**	**P-value**
rs9481067 (GG vs. AG + AA)	4.171 (2.332-7.431)	1.24E-06	4.747 (2.527-8.948)	1.24E-06
rs4837345 (TT vs. TC + CC)	4.721 (2.403-9.052)	4.00E-06	4.563 (2.242-9.107)	1.97E-05
kgp10457679 (CC vs. CT + TT)	4.721 (2.403-9.052)	4.00E-06	4.563 (2.242-9.107)	1.97E-05
kgp4136779 (TT vs. TC + CC)	4.721 (2.403-9.052)	4.00E-06	4.563 (2.242-9.107)	1.97E-05
rs1075650 (GG vs. AG + AA)	4.721 (2.403-9.052)	4.00E-06	4.563 (2.242-9.107)	1.97E-05
rs7314811 (CC vs. CT + TT)	4.586 (2.338-8.772)	5.66E-06	3.654 (1.746-7.425)	4.15E-04
rs6596805 (GG vs. AG + AA)	3.862 (2.139-6.908)	5.72E-06	3.683 (1.957-6.887)	4.51E-05
rs11047047 (GG vs. AG + AA)	3.734 (2.104-6.613)	5.92E-06	3.505 (1.908-6.426)	4.79E-05
rs1661281 (TT vs. TC + CC)	5.284 (2.518-10.764)	6.12E-06	5.403 (2.442-11.704)	2.17E-05
rs919001 (AA vs. AG + GG)	3.913 (2.134-7.077)	7.46E-06	3.151 (1.636-5.953)	4.66E-04

CI: confidence interval, OR: odds ratio.
OR compares the odds of having mucinous tumors in subgroup a to the odds of having mucinous tumors in subgroup b.
*Multivariable logistic regression model adjusting for the selected baseline characteristics listed in S5 Table.

S4 Table. Top ten most significant common SNPs identified under the univariable analyses and the subsequent multivariable analyses under the co-dominant genetic models.

	Univariable		Multivariable*	
**SNP ID (a vs. b)**	**OR (95% CI)**	**P-value**	**OR (95% CI)**	**P-value**
rs7314811 (CC vs. TT)	5.974 (2.803-12.805)	3.48E-06	4.788 (2.109-10.853)	1.63E-04
rs16907305 (AA vs. GG)	5.550 (2.611-11.857)	7.91E-06	4.505 (1.994-10.162)	2.66E-04
rs11216624 (AG vs. GG)	3.872 (2.092-7.050)	1.15E-05	3.326 (1.727-6.302)	2.57E-04
rs17712784 (AG vs. GG)	3.520 (1.988-6.243)	1.50E-05	3.304 (1.788-6.106)	1.28E-04
rs6573132 (AG vs. GG)	4.814 (2.308-9.722)	1.62E-05	5.183 (2.382-11.030)	2.27E-05
rs8019850 (TC vs. CC)	4.802 (2.302-9.699)	1.67E-05	5.011 (2.310-10.619)	3.07E-05
rs17093005 (TG vs. GG)	4.802 (2.302-9.699)	1.67E-05	5.098 (2.345-10.828)	2.66E-05
rs11656626 (GG vs. AA)	7.194 (2.866-17.642)	1.72E-05	7.156 (2.675-18.933)	6.94E-05
rs1189903 (AC vs. CC)	4.759 (2.284-9.589)	1.78E-05	4.952 (2.283-10.488)	3.47E-05
rs4779810 (TT vs. CC)	5.965 (2.608-13.499)	1.79E-05	4.286 (1.731-10.343)	1.30E-03

CI: confidence interval, OR: odds ratio.
OR compares the odds of having mucinous tumors in subgroup a to the odds of having mucinous tumors in subgroup b.
*Multivariable logistic regression model adjusting for the selected baseline characteristics listed in S5 Table.

S5 Table. Baseline characteristics selected through a stepwise variable selection method under the multivariable model.

**Characteristics**		**OR (95% CI)**	**P-value**
Age	≤60		
	60-65	2.29 (1.08-4.81)	0.018
	>65	1.19 (0.60-2.37)	0.611
Sex	Female		
	Male	0.58 (0.32-1.04)	0.067
Location	Colon		
	Rectum	0.45 (0.21-0.90)	0.031
Stage	I		
	II	4.41 (1.48-18.98)	0.018
	III	3.65 (1.18-16.02)	0.044
	IV	4.57 (1.18-22.43)	0.036
Grade	Well/moderately diff.		
	Poorly diff.	1.90 (0.70-4.54)	0.169

CI: confidence interval, diff.: differentiated.
OR: odds ratio (compares the odds of having mucinous tumors with the corresponding factor level to the odds of having mucinous tumors with the reference factor level).

S6 Table. AIC estimates under the multivariable models of common SNPs identified in the univariable analysis.

SNP ID	Initial Model	AIC				Plausible Model	P-value**
		A*	D*	R*	C*		
**rs9481067**	Recessive	322.4	336.5	318.4	320.2	Recessive	1.24E-06
**rs10511330**	Dominant	320.1	319.2	338.6	321.0	Dominant	1.40E-06
**rs16822593**	Dominant	320.2	319.3	338.6	321.1	Dominant	1.50E-06
**rs13019215**	Dominant	319.0	318.6	336.5	320.2	Dominant	8.20E-06
**rs12471607**	Dominant	318.9	318.6	336.4	320.1	Dominant	8.42E-06
**rs716897**	Dominant	323.4	321.2	337.0	323.0	Dominant	1.12E-05
**rs4843335**	Dominant	324.5	324.1	339.9	326.0	Dominant	1.48E-05
**rs11968293**	Dominant	327.2	322.4	338.9	324.4	Dominant	1.48E-05
**rs4837345**	Recessive	333.9	340.5	324.6	325.7	Recessive	1.97E-05
**kgp10457679**	Recessive	333.9	340.5	324.6	325.7	Recessive	1.97E-05
**kgp4136779**	Recessive	333.9	340.5	324.6	325.7	Recessive	1.97E-05
**rs1075650**	Recessive	334.1	340.6	324.6	325.6	Recessive	1.97E-05
**rs1661281**	Recessive	338.2	340.8	324.9	322.7	Recessive	2.17E-05
**rs6573132[1]**	Co-Dominant	334.3	328.9	339.0	325.0	Co-Dominant	2.27E-05
**rs205536**	Additive	321.3	330.3	326.0	323.3	Additive	2.34E-05
**rs17093005[1]**	Co-Dominant	334.2	328.9	338.8	325.1	Co-Dominant	2.66E-05
**rs11648965**	Additive	324.4	325.2	336.7	326.3	Additive	2.82E-05
**rs8019850[1]**	Co-Dominant	334.4	329.2	338.9	325.4	Co-Dominant	3.07E-05
**rs12915222**	Additive	323.5	327.9	331.9	325.5	Additive	3.19E-05
**rs1189903[1]**	Co-Dominant	328.0	323.4	334.2	320.9	Co-Dominant	3.47E-05
**rs6596805**	Recessive	331.2	339.6	325.3	327.1	Recessive	4.51E-05
**rs11159673**	Additive	325.5	329.4	331.1	326.8	Additive	4.53E-05
**rs11047047**	Recessive	328.4	338.3	325.2	327.1	Recessive	4.79E-05
**rs2384298**	Additive	323.3	324.0	333.6	324.5	Additive	5.14E-05
rs9809129	Dominant	319.6	321.4	335.0	321.0	Additive	6.52E-05
rs11656626[2]	Co-Dominant	331.5	337.6	326.6	328.3	Recessive	6.94E-05
**rs12956191**	Additive	325.5	330.0	331.2	327.4	Additive	8.18E-05
**rs17712784**	Dominant	327.6	326.5	340.7	328.5	Dominant	1.12E-04
rs7314811[2]	Co-Dominant	328.2	334.1	329.8	329.4	Additive	1.63E-04
rs16907305[2]	Co-Dominant	329.1	334.8	330.3	330.3	Additive	2.66E-04
**rs919001**	Recessive	330.4	337.4	329.7	331.0	Recessive	4.66E-04
rs11216624	Dominant	332.7	330.0	339.9	329.5	Co-Dominant	5.67E-04
rs4779810[2]	Co-Dominant	330.5	333.7	334.5	332.5	Additive	1.30E-03

*A: Additive, D: Dominant, R: Recessive, C: Co-dominant.
**p-value under the multivariable model based on the initial genetic model.
[1]: heterozygous genotype/major allele homozygous genotype.
[2]: minor allele homozygous genotype/major allele homozygous genotype.
The SNPs in bold were identified under their plausible genetic model.

S7 Table. Haploreg results for the top 10 SNPs in the common variant analysis.

SNP ID	chr	r2	D'	rs ID	GENCODE_name
**rs10819474**	9	0.92	0.96	rs4837345	PPP2R4
	9	0.91	0.96	rs192983	IER5L
	9	0.9	-0.97	rs944072	IER5L
	9	0.86	-0.99	rs10819473	IER5L
	9	0.94	-0.99	rs1966223	IER5L
	9	0.94	-0.99	rs1966222	IER5L
	9	0.99	1	rs12057089	IER5L
	9	1	1	rs10819474	IER5L
	9	1	1	rs10819475	IER5L
	9	1	1	rs419636	IER5L
	9	0.96	-1	rs12237274	IER5L
	9	0.9	-0.99	rs10739743	IER5L
	9	0.88	-0.99	rs4837346	IER5L
	9	0.94	-0.99	rs1556147	IER5L
	9	0.97	0.99	rs141780496	IER5L
	9	0.97	0.99	rs1075650	IER5L
	9	0.97	0.99	rs184457	IER5L
	9	0.93	0.97	rs882616	RP11-247A12.2
	9	0.93	-0.99	rs7034195	RP11-247A12.2
	9	0.91	-0.99	rs2005078	RP11-247A12.2
	9	0.83	-0.93	rs967497	RP11-247A12.2
	9	0.85	0.95	rs913264	RP11-247A12.2
	9	0.83	-0.93	rs4837347	RP11-247A12.2
	9	0.82	-0.92	rs7871824	RP11-247A12.2
**rs716897**	5	1	1	rs716897	RASGRF2
**rs4843335**	16	0.96	0.98	rs7500355	RP11-805I24.1
	16	1	1	rs4843335	RP11-805I24.1
**rs4837345**	9	0.8	0.94	rs9408986	PPP2R4
	9	0.8	0.94	rs71497442	PPP2R4
	9	0.8	0.94	rs4836641	PPP2R4
	9	0.8	0.94	rs1107329	PPP2R4
	9	1	1	rs4837345	PPP2R4
	9	0.97	1	rs192983	IER5L
	9	0.95	-1	rs944072	IER5L
	9	0.82	-0.97	rs10819473	IER5L
	9	0.9	-0.97	rs1966223	IER5L
	9	0.9	-0.97	rs1966222	IER5L
	9	0.91	0.95	rs12057089	IER5L
	9	0.92	0.96	rs10819474	IER5L
	9	0.92	0.96	rs10819475	IER5L
	9	0.92	0.96	rs419636	IER5L
	9	0.89	-0.97	rs12237274	IER5L
	9	0.83	-0.95	rs10739743	IER5L
	9	0.81	-0.95	rs4837346	IER5L
	9	0.87	-0.95	rs1556147	IER5L

	9	0.89	0.95	rs141780496	IER5L
	9	0.89	0.95	rs1075650	IER5L
	9	0.89	0.95	rs184457	IER5L
	9	0.86	0.93	rs882616	RP11-247A12.2
	9	0.87	-0.95	rs7034195	RP11-247A12.2
	9	0.84	-0.95	rs2005078	RP11-247A12.2
**rs9481067**	6	1	1	rs9481067	SLC22A16
	6	0.99	1	rs910399	SLC22A16
	6	1	1	rs761589	SLC22A16
**rs13019215**	2	1	1	rs13019215	CCDC141
	2	0.94	1	rs11680978	CCDC141
	2	0.93	1	rs150840830	CCDC141
	2	0.93	1	rs10930850	CCDC141
	2	0.92	0.99	rs12471607	CCDC141
**rs12471607**	2	0.92	0.99	rs13019215	CCDC141
	2	0.98	0.99	rs11680978	CCDC141
	2	0.98	0.99	rs150840830	CCDC141
	2	0.98	0.99	rs10930850	CCDC141
	2	1	1	rs12471607	CCDC141
**rs10511330**	3	0.92	0.96	rs16822588	ZBTB20
	3	0.95	0.99	rs16822593	ZBTB20
	3	0.97	0.99	rs73857113	ZBTB20
	3	1	1	rs6763403	ZBTB20
	3	1	1	rs10511330	ZBTB20
	3	0.94	0.97	rs73860251	ZBTB20
	3	0.91	0.97	rs16822606	ZBTB20
	3	0.91	0.97	rs7428451	ZBTB20
	3	0.9	0.96	rs6778079	ZBTB20
	3	0.9	0.96	rs6792964	ZBTB20
	3	0.9	0.96	rs6793257	ZBTB20
	3	0.9	0.96	rs57864250	ZBTB20
	3	0.9	0.96	rs73857603	ZBTB20
	3	0.9	0.96	rs6785090	ZBTB20
	3	0.9	0.96	rs73857605	ZBTB20
	3	0.9	0.96	rs2067756	ZBTB20
**rs16822593**	3	0.97	1	rs16822588	ZBTB20
	3	1	1	rs16822593	ZBTB20
	3	0.97	1	rs73857113	ZBTB20
	3	0.95	0.99	rs6763403	ZBTB20
	3	0.95	0.99	rs10511330	ZBTB20
	3	0.88	0.95	rs73860251	ZBTB20
	3	0.86	0.93	rs16822606	ZBTB20
	3	0.86	0.93	rs7428451	ZBTB20
	3	0.85	0.92	rs6778079	ZBTB20
	3	0.85	0.92	rs6792964	ZBTB20
	3	0.85	0.92	rs6793257	ZBTB20
	3	0.85	0.92	rs57864250	ZBTB20
	3	0.85	0.92	rs73857603	ZBTB20

	3	0.85	0.92	rs6785090	ZBTB20
	3	0.85	0.92	rs73857605	ZBTB20
	3	0.85	0.92	rs2067756	ZBTB20
**rs11968293**	6	0.9	0.98	rs10708664	SLC35F1
	6	0.88	0.99	rs6940985	SLC35F1
	6	1	1	rs11968293	SLC35F1
	6	1	1	rs1572226	SLC35F1
	6	0.85	0.97	rs72967533	16kb 3' of SLC35F1
	6	0.82	0.93	rs11153730	29kb 3' of SLC35F1

To retrieve the data, we used the default conditions and selected the CEU (Caucasian) as the population.
Chr: chromosome, r2: correlation coefficient,
D': ratio of given and min/max coefficient of linkage disequilibrium depending on allele frequencies.

S8 Table. Proteins which have reported evidence of binding to the genomic region in which kgp10457679 resides (extracted from RegulomeDB).

Location	Bound Protein
chr9:131930083-131930707	PHF8
chr9:131930124-131930734	POLR2A
chr9:131930177-131930837	SMARCB1
chr9:131930235-131930775	EP300
chr9:131930398-131930728	EP300
chr9:131930394-131930710	EP300
chr9:131930310-131930710	FOS
chr9:131930295-131930751	GTF2F1
chr9:131930295-131930751	JUND
chr9:131930321-131930661	JUN
chr9:131930369-131930709	JUN
chr9:131930291-131930687	MAX
chr9:131930308-131930772	POLR2A
chr9:131930314-131930520	POLR2A
chr9:131930373-131930729	RCOR1
chr9:131930379-131930709	RFX5
chr9:131930359-131930715	TAF1
chr9:131930275-131930711	UBTF
chr9:131930369-131930679	IRF1
chr9:131930380-131930700	JUND
chr9:131930343-131930699	MAX
chr9:131930332-131930702	MAZ
chr9:131930374-131930684	MYC
chr9:131930403-131930699	RCOR1
chr9:131930351-131930667	YY1
chr9:131930381-131930677	JUN
chr9:131930388-131930672	FOS
chr9:131930392-131930656	CEBPB
chr9:131930395-131930671	JUND
chr9:131930405-131930655	FOS
chr9:131930406-131930666	FOSL2
chr9:131930420-131930656	BACH1
chr9:131930423-131930659	ATF3
chr9:131930419-131930649	JUN
chr9:131930417-131930627	MAFF
chr9:131930376-131930666	USF2
chr9:131930451-131930567	MAFK
chr9:131930484-131930584	FOS
chr9:131930489-131930574	FOS
chr9:131930462-131930577	NFE2
chr9:131930433-131930605	FOSL2
chr9:131930433-131930610	MAFF
chr9:131930436-131930602	MAFK

chr9:131930442-131930598	MAFK
chr9:131930432-131930604	MAFK
chr9:131930210-131930654	POLR2A
chr9:131930433-131930663	FOSL1
chr9:131930443-131930622	BACH1
chr9:131930438-131930615	MAFK
